# Urban Infrastructures of Care: Buses as Resources for Recovery and Mental Wellbeing

**DOI:** 10.1111/1467-9566.70278

**Published:** 2026-10-20

**Authors:** Marc Winz, Ola Söderström, Aurora Ruggeri, Jérôme Favrod, Jules Brischoux, Anastasie Jordan, Lilith Abrahamyan Empson, Philippe Conus

**Affiliations:** ^1^ Institute of Geography University of Neuchâtel Neuchâtel Switzerland; ^2^ Treatment and Early Intervention in Psychosis Program (TIPP), Service of General Psychiatry Department of Psychiatry Lausanne University Hospital (CHUV) Lausanne Switzerland; ^3^ Swiss Society of Social Psychiatry (So‐Psy) Lausanne Switzerland

## Abstract

Although transport infrastructures are increasingly recognised as determinants of urban wellbeing, their role in trajectories of becoming well and becoming ill has received little empirical and theoretical attention, particularly in serious mental ill‐health. This paper examines how bus infrastructures function as everyday resources and obstacles for people living with psychosis. Drawing on qualitative research with young people recovering from early psychosis in Lausanne, Switzerland, we examine the socio‐material and affective dimensions of bus mobility through relational geographical perspectives on health and place, mobility studies and work on infrastructures of care. We show how buses can function as affective lifelines, anchors of autonomy and independence and gateways to formal and informal resources for wellbeing, supporting recovery and re‐engagement with the city. Buses can also entail sensory overstimulation, proximity to others, perceived surveillance, anxiety and financial constraints. Our analysis highlights how mobility capacities are relationally constituted through encounters with urban infrastructures: buses can cultivate or undermine capacities to engage with the city. We argue that buses should therefore be understood not simply as transport services or conduits to therapeutic resources, but as ordinary infrastructures of care and enabling places through which recovery is enacted in urban life.

## 1 Introduction

Public transportation systems are essential to the daily functioning of metropolitan areas. Beyond serving as a mere means of movement, they constitute a vital gateway to participation in urban life. Yet using public transportation is far from straightforward. A seemingly ‘simple’ bus journey requires navigating a complex sequence of physical, cognitive and social tasks—before, during and after the trip. These include locating and accessing infrastructure, identifying and boarding the correct service, managing ticketing, monitoring cues throughout the ride and navigating to the final destination of the journey, all while managing interpersonal communication, adapting to social interactions and coping with potential disruptions (Mackett 2017). Unpacking a bus journey in this way reveals the multifaceted social, material and affective dimensions of everyday bus mobility. Because bus travel is often taken for granted as unproblematic, its lived experience has received limited scholarly attention (Joelsson et al. 2025). Therefore, although interest in the links between public transport, mental health and care is growing, these connections remain underexamined and appear, for example, only marginally—if at all—in recent studies and reviews on urban living and mental health, whether framed as risk or protective factors (B. E. Evans et al. 2023; Gruebner et al. 2017; Okkels et al. 2018; Pykett et al. 2023; Xu et al. 2023). Thus, the role transport infrastructures play in shaping the nexus between urban life and mental health is still insufficiently understood—let alone their place within trajectories of becoming ill and becoming well; and this gap is even more pronounced in the context of serious mental ill‐health (Mackett 2021; Melis et al. 2015).

Nonetheless, accessible public transportation has recently been proposed as a means to enhance citizens' mental health and wellbeing and mitigate urban risks for mental health in a position paper by the European Psychiatric Association, which advocates for measures such as electric public transportation, car‐free zones and separated bike and scooter zones (Misiak et al. 2025). Hence, although public transportation may hold significant potential for co‐shaping wellbeing and mental health, realising this potential requires more detailed, experiential knowledge of how mental health and public transport are entangled in everyday urban life. Without such insight, fine‐grained, contextually grounded and relevant recommendations remain difficult to formulate. This article contributes to advancing this understanding.

In the following, we first review relevant literature on the entanglement of (urban) mental health, public transportation and buses. Second, we elaborate a theoretical approach anchored in a relational geographical perspective on health and place (Duff 2012, 2016; Söderström et al. 2016), mobility studies (Kaufmann et al. 2004) and recent proposals framing buses as infrastructures of care (Joelsson et al. 2025; Stratford and Byrne 2024) and enabling infrastructures (Park et al. 2024). We then present our methodology and our results, interpreting the latter in the light of our theoretical proposal. Our empirical analysis reveals the dynamic interplay between public bus infrastructure and proactive processes of becoming well in and with the city in the context of psychosis. By attending to the socio‐material‐affective entanglements of bus mobility for persons living with psychosis, our paper reframes the bus as an infrastructure of care. More broadly, in reclaiming urban public transport as a resource for wellbeing, this article envisages cities and their infrastructures as active producers of mental health and recovery trajectories.

## 2 Literature Review: Buses, Public Transportation and Mental Health

Studies exploring the relationship between public transportation and health have either focused on population‐wide effects or on specific vulnerable groups. Strong evidence indicates that public transportation benefits physical health, in part because most trips involve walking (UNECE 2024), a key dimension of active mobility (J. T. Evans et al. 2024; Patterson et al. 2020). Its relation to mental health is, however, less well understood. Population‐level studies have reported mixed findings, with some suggesting that greater public transport use is associated with better mental health (Ferenchak and Katirai 2015; Martin et al. 2014), whereas others find no significant association (Bouck et al. 2025; Posner et al. 2018). Overall, evidence remains mixed and is limited in its capacity to disentangle the effects of public transport use itself from those of the destinations themselves or other contextual factors associated with travel (Scrivano et al. 2024). These findings contrast with users' perceptions. In a UK survey of 243 public transport users, Posner et al. (2018) found that nearly all participants believed transport systems had negative effects on mental health, whereas none perceived them as having exclusively positive effects.

With regard to vulnerable groups, most existing studies, reports^1^ or literature reviews adopt a broad and unspecific approach to mental health (Cooper et al. 2019; Faulkner et al. 2023; Garg et al. 2022; Mackett 2021; Nielsen 2021; Posner et al. 2018), except with regard to older adults (see Dilian et al. 2024) and those living with dementia (see Lanthier‐Labonté et al. 2024), as well as persons living with autism spectrum disorder and attention deficit hyperactivity disorder (Bolic Baric et al. 2025). Common to this body of work on public transport and vulnerability is a focus on barriers, recurring obstacles negatively affecting wellbeing and mental health, as well as impairing the use of public transport. Although public transportation mediates mental health, mental health conditions also, in turn, affect people's ability to use public transport (Dilian et al. 2024; Mackett 2021). Travelling by public transport requires a range of cognitive, social and financial resources and skills, which may be shaped by mental health difficulties—thereby creating barriers to the use of public transport.

Social interactions represent a significant source of difficulty. Encounters and interactions with transport staff and other passengers can be an important source of distress (Faulkner et al. 2023; Nielsen 2021; Posner et al. 2018), particularly when this interaction is characterised by misunderstandings or (auto)stigmatisation. Faulkner et al. (2023) highlight the cumulative and intersectional nature of these challenges, as the experience of transport stress is intensified when multiple vulnerabilities—such as gender, race and mental health status—intersect. Spatial and sensory factors—such as crowding (Faulkner et al. 2023; Nielsen 2021; Posner et al. 2018), noise and the experience of limited or disputed space (Faulkner et al. 2023)—are repeatedly identified as key sources of discomfort contributing to sensory overload and feelings of anxiety (Faulkner et al. 2023; Nielsen 2021; Posner et al. 2018). Information (or lack thereof) and journey‐planning challenges constitute another recurrent theme. Difficulties in accessing reliable, real‐time information, service disruption and problems related to wayfinding and navigating unfamiliar routes can heighten uncertainty and anxiety (Nielsen 2021; Posner et al. 2018). The lack of adequate support to help manage uncertainty or distress is a supplementary source of anxiety (Faulkner et al. 2023; Posner et al. 2018). Accessibility and reliability of services—such as the frequency, distance to stops and punctuality—further constrain mobility. Unreliable or infrequent services amplify time pressure and unpredictability, which are particularly stressful for individuals managing mental health conditions (Faulkner et al. 2023; Nielsen 2021; Posner et al. 2018). Finally, financial barriers also play a crucial role, limiting access to transport options (Nielsen 2021) and thereby reinforcing immobility and social exclusion.

These barriers underscore how public transport infrastructures remain poorly adapted to the needs of people with mental health difficulties, revealing a form of (mobility) injustice. They also overlap with sources of urban stress identified in previous work—such as sensory overload, unsolicited social contact and disruptions to fluid mobility—which contribute to avoiding city centres after a first episode of psychosis (Söderström et al. 2016). Although persons living with psychosis have been included in some of the above‐reviewed literature, to the best of our knowledge, no study has focused on their lived experiences of using public transportation. Existing work, however, relates the (non‐)use of public transportation to symptomatology. For instance, reduced mobility patterns and difficulties in mobility have been considered a marker of negative symptoms in psychosis (Depp et al. 2019; Viertiö et al. 2009). Conversely, greater mobility has been associated with greater connection to community life (Depp et al. 2019). In terms of access to healthcare, it has been found that greater distance to public transportation predicts a longer duration of untreated psychosis, independently of proximity to clinics (Malinowski et al. 2023)^2^. Although we must be careful not to overstate its relative weight among other determinants, this point is nonetheless noteworthy when considered alongside evidence showing that the duration of untreated psychosis can shape real‐life outcomes of psychotic disorders (Golay et al. 2023).

Overall, the literature suggests that the relationship between mental health and public transportation is ambivalent and remains insufficiently understood. Whereas barriers to using public transport have been rather well documented, the positive dimensions and the affective experience of public transportation have received little attention. More broadly, the role of public transportation within urban mental health is underexplored, despite evidence that it can be crucial for accessing help in the context of psychosis. There is a notable lack of research examining how public transportation intersects with processes of recovery and becoming well in the city, whether for people experiencing psychosis or for mental health more generally. Our paper contributes to reducing this research gap by theoretically framing buses as infrastructures of care (Stratford and Byrne 2024) and empirically exploring how participants living with a diagnosis of psychosis experience buses.

## 3 Theoretical Framework: From Therapeutic Places to Enabling Infrastructures

Place‐based approaches to wellbeing and mental health have gained substantial traction since the late twentieth century, particularly after Gesler’s (1992) agenda‐setting formulation of *therapeutic landscapes*. This work created significant momentum and provided fertile ground for scholars to further elaborate conceptual tools for examining the entanglements of place, health and trajectories of becoming ill or well. Critically building on Gesler's work, two influential extensions have been proposed: *landscapes of care* (Milligan and Wiles 2010) and *enabling places* (Duff 2012, 2016), adopting a relational and processual perspective, emphasising the practices, meanings and affective relations through which people produce their therapeutic potential in everyday life. The notion of *landscape of care* (Milligan and Wiles 2010) builds on work on therapeutic landscapes to reframe care as a networked practice where care unfolds across multiple settings—from homes and neighbourhoods to formal institutions—and at various scales, while also being shaped by power dynamics. The notion of ‘enabling places’ revisits these frameworks by focussing on the emergent capacities of place to support (or disable) forms of wellbeing, health and recovery in mundane places. With ‘enabling places’, Duff (2012, 2016) adds an analytical vocabulary distinguishing social, material and affective resources actively produced in our everyday relations to places. Social resources correspond to the processes and interactions through which individuals create and maintain social connections. Material resources encompass the diverse objects, assets and benefits that circulate within and between local social and economic networks, along with the material opportunities and affordances present in different urban environments. Affective resources involve emotions, memories and attachments embedded in place, as well as the felt sense of place. In a similar vein, but with more focus on individuals, Doroud et al. (2018) suggest four dimensions through which place‐based relations foster wellbeing and recovery: *Being* (feeling safe, grounded and present), *Doing* (engaging in meaningful activities), *Becoming* (orienting towards a hoped‐for future self) and *Belonging* (experiencing connection to others and to place). These frameworks ‘[…] are useful to capture the interaction between resources, processes, ideas and place in the provision and experience of care, putting care firmly *in place*, as argued by Wiles in a more recent review on landscapes of care (2024, 371, emphasis in original). However, this citation also highlights a limitation: as these concepts *firmly* situate care *in place*, they may overlook the space‐times between places, the mobile ways of inhabiting space when walking, driving, biking or taking the bus^3^.

To include those space‐times, our analysis also draws on debates in transport and mobility studies and their concerns with issues of justice and (in)equality. Whereas transport justice has primarily focused on the equitable distribution of transport infrastructures and services, mobility justice broadens the lens to consider how power relations, recognition and structural inequalities shape people's opportunities to move and participate in everyday life (Sheller 2018; Verlinghieri and Schwanen 2020). Within mobility studies, the concept of motility (Kaufmann et al. 2004) offers a particularly useful lens for examining how these structural inequalities translate into differential capacities to engage with mobility in everyday life. Motility conceptualises mobility not as movement itself but as a socially produced capital, constituted through a combination of differential access, competences and appropriation (Kaufmann et al. 2004). Building on these perspectives, we approach mobility capacities as relational achievements emerging through encounters with urban infrastructures. Although transport justice rightly draws attention to the equitable distribution of transport systems, our analysis suggests that justice also depends on whether and how these infrastructures cultivate the capacities through which people can benefit from mobility. Such understanding directs attention to the embodied, affective and relational dimensions of mobility foregrounded by cultural geographers (Bissell 2018; Cresswell 2006; Merriman 2012). This body of work shows that commuting is not merely a functional act of travelling but an embodied, affective and relational practice. Drawing on Bissell (2018), we understand buses as socio‐material infrastructures that engender a range of ‘affective intensities’, augmenting or diminishing people's capacities to act, relate with others and engage with the city. Here, affect refers not simply to feelings or emotions but to the relational forces emerging through encounters between bodies, infrastructures and urban environments that shape capacities for action and engagement. Whereas Duff conceptualises affective atmospheres as resources through which places become enabling, Bissell foregrounds the affective intensities generated through movement itself. Together, these perspectives allow us to understand buses not only as enabling places in motion but also as environments in which capacities for recovery are continually augmented or diminished through everyday encounters. This understanding resonates with recent calls to examine how buses and public transport support everyday practices of care and can be understood as ‘infrastructures of care’ (Stratford and Byrne 2024) and ‘enabling infrastructures’ (Joelsson et al. 2025) whose significance extends beyond the provision of mobility itself. This interpretation also aligns with recent efforts to reconceptualise urban infrastructures through the lens of the urban commons, highlighting their capacity to cultivate conviviality and broaden networks of care (Park et al. 2024).

Weaving together emplaced approaches to health and embodied approaches to urban commuting, we approach buses as ordinary urban places where care, wellbeing and recovery are cultivated through the everyday experience of moving with others. Mobility infrastructures are thus more than conduits connecting origins and destinations: they are places in which care, wellbeing and recovery may themselves be enacted. This framework allows examining the experience of bus travel and bus infrastructures as intertwined social, material and affective relations, and to explore how public transportation contributes to processes of becoming ill or well in the city. Such an approach challenges understandings of urban mobility infrastructures as a passive backdrop to ill‐health, instead foregrounding the complex ways in which they actively participate in shaping health. Rather than treating mobility infrastructures as resources to which people have differential access, we conceptualise them as relational environments that actively cultivate or undermine the capacities through which recovery is enacted in everyday urban life.

## 4 Methods

### 4.1 Research Context and Analytical Scope

This paper draws on empirical material generated within larger participatory action research based on a living lab approach, which seeks to co‐design and evaluate ways of cultivating an urban environment that supports recovery from psychosis. Here, we develop an interpretive account of one recurring theme that emerged during the exploratory phase of the living lab, which was dedicated to developing an in‐depth understanding of participants' everyday urban experiences, rather than reporting on the whole project. Our analysis draws on qualitative material documenting everyday urban practices and identifying spatial, social, affective and institutional resources—as well as obstacles—that shape psychiatric service users' experience of the city. Across these accounts, the bus emerged as a significant resource in variegated ways and at different moments in participants' recovery trajectories.

Although this exploratory phase of our work also identified commercially oriented third places, public spaces, cultural institutions and green spaces as important sites of urban recovery, addressing these domains lies beyond the scope of the present paper. We focus instead on buses as a particularly revealing case through which to examine how an urban infrastructure becomes a central and active resource for recovery, shaping trajectories of becoming well in everyday life. The living lab subsequently moved into phases of co‐elaboration, experimentation and upscaling (Ballon et al. 2018; J. Evans et al. 2018; Malmberg and Vaittinen 2017) during which interventions were co‐designed and tested across several of these domains, including public transport. Although these later phases are not the empirical focus of this paper, they provide an important backdrop for the discussion. The fine‐grained ethnographic account of buses as infrastructures of care we develop in this paper leads us to concluding remarks on how such insights might inform the design of urban interventions for mental health.

The study was carried out in partnership with the outpatient psychiatry department of Lausanne's University Hospital, specifically within the Treatment and Early Intervention in Psychosis Programme. Ten participants were recruited among programme service users who met the following inclusion criteria: aged between 18 and 35 years; presenting psychotic symptoms above the threshold as defined by the CAARMS instrument; and having received less than 6 months of antipsychotic medication prior to enrolment. To align with the study's spatial focus, participants were also required to live in, or frequently use, the central districts of the city. We ensured that participation would not interfere with participants' wellbeing or clinical follow‐up. Participants received financial compensation. Ethical approval was obtained from the Cantonal Research Ethics Committee (Insert Ethics information). All participants gave written consent prior to participation.

### 4.2 Data Collection

Data were primarily produced through walking interviews (J. Evans and Jones 2011), as mobile in situ interviews are particularly well suited to examine how people relate to and experience their urban environments in health research (Carpiano 2009; J. Evans and Jones 2011; Foley et al. 2020). Fieldwork was conducted by a multidisciplinary team composed of a psychiatrist, a postdoctoral and a doctoral researcher in human geography and a co‐researcher with lived experience of psychosis. Walking interviews were carried out in pairs (researcher–participant), whereas the co‐researcher joined one pair to form a trio, offering additional experiential perspective and participating in the discussion. The walks were organised along four predefined routes designed to include different types of urban settings—commercial streets, green areas, transportation nodes and quieter residential segments. The choice to predetermine the route rather than have participants lead was informed by previous collaborative experience, which showed that not having to lead the walk and decide where to go felt comforting for the participants. The choice was also motivated by our objective, which was to capture a variegated sample of the city's different ambiances (Thibaud 2011, 2015). Although routes were primarily conducted on foot, portions were occasionally completed by bus for practical reasons.

During the walks, participants were invited to reflect on their experiences of different places throughout their recovery trajectories. In addition, participants were asked to take photos of significant settings. The primary objective was to map resources and barriers to wellbeing and recovery. Walks proceeded at a comfortable pace, with breaks when needed, and lasted about 1 h. At the end of each session, participants and researchers stopped in a nearby café to discuss impressions of the walk and annotate printed maps with notes or sketches, for another 30–60 min. Each participant took part in three to four walks. Once all 32 walks were completed, the full group gathered for a collective discussion workshop to reflect on emerging patterns. The dataset thus comprises recorded interviews, participant photographs, annotated maps, the group discussion and reflexive discussions with the co‐researcher with lived experience. Together, these materials provide complementary accounts of how urban environments and infrastructures enable or constrain recovery.

### 4.3 Data Analysis

Interviews were transcribed and imported into MAXQDA (MX.24) for analysis, together with participants' photographs. Analysis followed an abductive approach, in which ‘existing theory and literature is applied in tandem with the raw data to explain the patterns and story behind the data’ (Thompson 2022, 1411). An initial theory‐driven codebook was developed drawing on the concept of *enabling places* (Duff 2012) and on a meta‐ethnographic framework addressing the role of place in mental health recovery (Doroud et al. 2018), with additional codes developed inductively throughout the analytical process. Although the analysis draws on the broader corpus generated during the exploratory phase, this paper is structured around the experiences of two participants, Greta and Bazile. These accounts were identified as particularly analytically productive because they provided rich insights into an underexplored dimension of the literature: the role of buses in processes of becoming well.

## 5 Findings: Buses and Bus Infrastructures as Resources for Recovery and Wellbeing

The following findings highlight how buses cultivate distinct capacities for engaging with the city, showing how buses become infrastructures of care in different ways across recovery trajectories. Although Greta's and Bazile's accounts provide the central thread of the analysis, they are interpreted in relation to the broader empirical corpus, including the experiences of other participants whose narratives complement and nuance the findings.

### 5.1 Navigating the City After Psychosis, Shielded by the Bus

Greta (30 years old) was very motivated to participate in our research when she heard of it through her case manager. She later explained that she saw this as a unique opportunity to contribute to potentially easing difficulties experienced by others who share her struggles. During our fieldwork, she was working part‐time at an independent cinema and preparing to resume her university studies. She thus was rather busy but managed to fit her participation into her schedule, as it was very important to her. During the walks and conversations, Greta shared that her entire way of moving through the city changed with her experience of psychosis. The bus only really came to play a role in her life with the onset of this experience. In her life ‘before’, she told us, she used to like to walk and walked a lot, on a daily basis. Then, during the period shortly before the onset of psychosis, walking became compulsive as her way of trying to cope with her symptoms.

Greta […] And then, when I had the decompensation – well, a little before the decompensation – I was walking all the time. I never took public transport; I just kept walking, running, walking, walking. I think I was walking four to 5 h straight every day

MW Really?

Greta Yeah. I was trying to escape a lot of what I was hearing. During the decompensation, I stopped eating, I stopped sleeping, and I stopped drinking. So, yeah, all I did was walk and run at night

She went on to explain that these long hours spent walking generated a form of ‘trauma’, to use her words, in which walking in the city became intimately associated with hearing voices. Her mobility routines changed drastically in her ‘life after decompensation’, after she reached out for help and spent some time in the hospital. When she returned home, the bus came to play a central role in her everyday life, shaping both her relationship with the city and her recovery journey. During that period, Greta withdrew almost completely, spending nearly a year at home, going out only occasionally—mostly to attend her outpatient visits. To do so, instead of walking, Greta started using the bus.

Greta […] And also, something I wanted to say from the start is: thankfully there's public transportation, because with the illness, walking was really difficult. Honestly, it's really great, that's why I have my bus pass. Before, I never took the bus; I walked all the time. But after the decompensation, I started taking the bus more and more often because it feels safer for me

LA Safer in what way?

Greta In the sense… well, I had this thing where I couldn't hear the voices. I always had this trauma from walking and hearing things, whereas the bus is enclosed, so I felt more secure

LA Okay. Because of the voices?

Greta Yeah, but even when I didn't hear them anymore, that trauma was still there. Especially after leaving the hospital, it really helped me get to my TIPP [the outpatient consultation] appointments. For example, the bus stops right next to the CHUV [university hospital] offices—that's amazing! <enthusiastic>

LA So the bus was your safe space?

Greta Oh yeah, definitely. I'd get on a bus, take another one, and get off at TIPP, then do the same on the way back. Honestly, without that, I'm not sure I would have gone out. And sometimes I'd think, ‘Okay, just a little walk.’ I think Lausanne's public transport system, aside from a couple of issues, is really good. It's just too expensive, but otherwise it's fine [laughs]

Her description pictures the bus as having a buffering effect, a space where she felt reassured and safe, shielding her from the voices she had been hearing in the street while walking. Moreover, occasionally it encouraged her to walk a few steps, although it felt difficult. This connects with a point Greta made on various occasions during the interviews: she described using the bus as energy‐saving. With her psychosis, walking became increasingly demanding, both mentally and physically, leaving her with less energy for other activities. Hence, using the bus as an alternative during recovery allowed her to manage her energy more effectively and gave her the strength to undertake meaningful activities she wouldn't have been able to do otherwise.

Thus, buses provided her with a means to access both formal resources and informal resources for recovery. On one side, it helped her to attend her outpatient appointments, because, as she puts it: ‘*If I had had to walk from my house to the TIPP, I don't know if I would have done it*’. On the other hand, when passing in front of a public library, Greta explained that this was her favourite, ‘above all, because it's actually quite close to all the buses. So, when I wasn't feeling well, I could just come by bus, sit down, find lots of books, and then quickly go home’. However, it also encouraged her, as mentioned, to walk a few steps in a process of self‐growth, as well as it allowed her to stay connected with some of her friends during these troubled times, when she forced herself to leave home to catch up with friends in town. Thus, beyond providing access to healthcare and other urban opportunities, the bus appears as a threshold space through which she negotiates her engagement with the city, with others and with herself. Buses mediate the interplay between places, people and resources through which recovery is continually negotiated in daily life: even if the symptoms are no longer present, the bus remains a safe space for Greta, as walking in the street is still closely associated with memories of her ‘trauma’. This experience continues to influence Greta's mobility patterns, as she remains a frequent bus user. It is important to notice that the bus came to play this important role in her recovery process because, as she explains, bus riding was not associated in her case with social anxiety. On the contrary, the enclosed space of the bus was reassuring. This is an important point of contrast compared to the experience of other participants in our study, as we discuss below.

### 5.2 Bus Infrastructures as an Affective Lifeline

From the very beginning, Bazile (24 years old) was, like Greta, rather enthusiastic about participating in the research. He was the second participant we recruited, and he joined every walking interview and also nearly all the workshops we organised during the research. During the collective workshops, Bazile participated very actively in the discussion. He was often the first to speak, breaking the ice when other participants were more hesitant. Bazile has a remarkably detailed knowledge of the bus network—the routes, schedules and location of stops across the city. The transport system itself seemed to serve as a set of landmarks through which he oriented himself in the urban environment. At the time of fieldwork, Lausanne was ongoing major urban transformations, which also affected the bus network, and occasionally disrupted his ease and confidence in using buses.

For Bazile, buses played a particularly significant role in the period after his stay in hospital, complementing Greta's experiences in a distinct yet related manner. He describes the hospital environment as largely devoid of sensory and social stimulation, a stark contrast to the richness and intensity of urban life. As a result, time spent in the hospital has a desensitising effect, as he explains, leaving him temporarily unaccustomed to the sensory and social demands of the city. Once he returns home after discharge, this feeds a transient period of city avoidance (Conus et al. 2019) at first.

BAZILE Well, the city, I tend to avoid it when I get out [of the hospital]… That's why I walk along the lakeshore at different hours. If I'm not feeling really up to it, I'll rather go in the morning. Once I manage to go farther and tolerate… let's say the movement, the noise, the crowd, the smells—it allows me, let's say, to get back on track progressively

MW So you're really, little by little, regaining space and making it yours by…

BAZILE No, it's not even about that—it's just about getting used to it again. Because the hospital is a space… yeah, it's… everything is controlled. There's never a crowd moving around, no cars passing every 10 s, so of course it's different. And, well, since you go to the hospital either to strongly reduce stimuli or to start taking them in again all at once, I try to do it gradually, step by step

MW Right, so and with other spaces or services, you go at specific times—like the train station, there are places you avoid depending on the city's rhythms, would you say?

BAZILE I'd say that… yeah, for the first 2 weeks, 10 days or so, I hardly ever leave my apartment. But… it's not even that I couldn't manage to—it's just that… you're in such a state of… I don't even know how to explain—you're really drained, completely out of energy. And so, yeah, if I didn't make the effort to go to the lakeshore, I think I could stay like that for months, and that… I couldn't allow myself to

After each of his hospitalisations, going to the outpatient appointments is for Bazile the only moment when he leaves home for a few weeks, and he always uses the bus to do so. Although these appointments in town provide Bazile with a routine and compel him to engage with life outside, he then also adopts a more personal strategy to rebuild the capacity to be an urbanite. Bazile's progressive, gradual and groping re‐engagement with city life begins with walks along the quiet lakeshore and progressively moves towards the busier city centre, as he becomes more comfortable with the increasing intensity of sensory stimulation, movement and social presence. This process takes several weeks, and the bus plays a central but paradoxical role in it, as Bazile does not actually ride the bus:

BAZILE [… ]I think it was two or three years ago, they installed a new line, number 24, that runs, yeah, all along the lakeshore. It's great, so it shouldn't be removed

AR It's great because it lets you travel all along the lake?

BAZILE Especially when I go for walks and things aren't going so well, well, I can quickly take the bus, and get back home at full speed… a bit faster

AR Yeah, okay

BAZILE And especially when I'm trying to get used to things again, after coming out of the hospital, well, it's a challenge each time to go… to try going a bit farther

AR And this bus works well for that?

BAZILE Yes, it's perfect for that

For Bazile, after discharge, the bus accompanies this process of *re‐acclimatisation* to the city's stimuli and his progressive approach to the city centre. He does not ride the bus during this process, shielded from the wider urban space as does Greta. He walks. On purpose, as he is seeking to actively encounter these stimuli, in order to get used to them. However, he walks along a specific and familiar bus route—knowing the schedules, stops and levels of crowding at different times of the day. Here, the bus infrastructure functions as an *affective lifeline*, offering him a sense of safety and reassurance: if he begins to feel unwell, he knows he can board the next bus and get home quickly. This sense of security is anchored in the bus infrastructure as a socio‐material and affective resource he can rely on at any moment, simply by boarding the next and going home as quickly as possible. Bazile's knowledge, his testimony and use of the public transportation system are particularly telling, as they highlight the interplay between accessibility, competences and appropriation foregrounded by the notion of motility (Kaufmann et al. 2004). The mere presence of the bus line functions as an affordance through which Bazile enacts active and conscious practices of recovery that otherwise would not be possible. In this sense, bus infrastructures contribute to what has been conceptualised as processes of becoming—nurtured by hope and self‐growth—in the broader trajectory of becoming well in place (Doroud et al. 2018). Without actually riding the bus, bus infrastructures function as ‘enabling infrastructures’ (Park et al. 2024), thereby contributing in unexpected ways to processes of becoming well in and with the city.

## 6 Discussion and Conclusion

Although every ill‐health trajectory is idiosyncratic—and the ways people draw on everyday social, material and affective resources vary—Greta's and Bazile's accounts denote broader patterns regarding the entanglement between bus infrastructures and urban mental health. Their narratives provide valuable insights into how buses shape processes of becoming well after psychosis, illuminating an underexamined dimension of a literature that has focused primarily on barriers to mobility and urban participation in the context of mental health. Greta's account shows how buses support recovery through buffering symptoms, affording energy management and allowing engagement, beyond access to both informal and formal resources. Bazile, for his part, demonstrates how bus infrastructures provide confidence, a feeling of control over space and orientational cues, and foster gradual reengagement with the city in a process of self‐growth, without even riding the bus. These are not two different functions of buses but two expressions of the same process: rather than merely facilitating access to therapeutic resources, buses provide different capacities for recovery through different affective relationships with the city. These findings invite a reconsideration of how place‐based approaches conceptualise recovery, extending attention from relatively stable therapeutic places to the ordinary mobility infrastructures through which recovery is enacted in everyday urban life.

As a means of transport, buses dynamically weave together the rather fixed ‘enabling places’ (Duff 2016) that have been explored in the literature to form a ‘landscape of care’ (Milligan and Wiles 2010) through movement across places. However, although buses offer a medium for engaging with enabling places across the city, they become themselves enabling places in motion and ‘enabling infrastructures’ (Park et al. 2024) as they afford subtle affective potentialities for becoming well in the city. Whether intensively used or merely present in the background as a reassuring potential means of transport, they constitute socio‐material environments through which capacities for recovery are continually cultivated through everyday encounters. Thus, more than a simple means of transport, buses offer, for example, a valuable source of independence and agency, personal growth, hope and social connection, all considered essential components of place‐based processes of recovery (Doroud et al. 2018). At the same time, our findings caution against treating buses as inherently enabling.

The experiences of Greta and Bazile we chose to focus on in this article should not overshadow the barriers and obstacles that can come with bus journeys and public transportation we discussed in the literature review (Faulkner et al. 2023; Nielsen 2021; Posner et al. 2018), and some of which we also encountered. As one participant reflected, the very act of taking the bus to reach a place perceived as beneficial can be a hindrance, when the stress and unease of the journey outweigh the restorative effects of leaving home. This brings to the foreground that buses are not enabling per se: challenges and barriers are associated with this means of transport. The bus is a confined space where co‐presence and proximity to others may be experienced as uncomfortable or distressing—especially by those living with social anxiety. Buses are also spaces where some participants felt the gaze of others more intensely, to the point that they may avoid taking the bus altogether or only do so with great effort. Jules, the co‐researcher and co‐author with lived experience, explains how the fear of not behaving according to expected social norms, not blending in and being perceived as different can be enhanced in enclosed spaces, such as public transport, cafés and restaurants. The absence of an immediate escape can reinforce feelings of confinement and unease. These difficulties overlap partly with social barriers identified in studies approaching the link between public transport and persons living with mental health difficulties through broad and undifferentiated categories (Faulkner et al. 2023; Nielsen 2021; Posner et al. 2018), as well as findings with urban stress and psychosis (Söderström et al. 2016). Jules further explains that overhearing other passengers' conversations and repeated unwanted eye contact can lead to the impression that others are talking about you, potentially nurturing delusional thoughts and overinterpretations. Public transportation is therefore characterised by a fundamental ambivalence: it can both facilitate and hinder access to the city, and support or impede subtle processes and endeavours of getting better—ultimately contributing to ill‐health.

The contrasting experiences described by participants highlight that what matters is not the bus itself but the affective encounters that emerge within it. The same infrastructure may therefore cultivate or undermine recovery depending on how these encounters unfold. The affective intensities afforded by buses (Bissell 2018)—which augment or diminish people's agency—are central in this perspective. It highlights buses as a central element in the ‘intensities of engagement’ used by Codeluppi (2019) to describe the varying degrees to which young persons living with psychosis interact with their everyday urban environment during the process of recovery, and how this engagement fluctuates throughout the process. Understanding buses through these intensities also helps us understand why and how they can be understood as infrastructures of care, alongside evidence linking mobility to symptomatology (Depp et al. 2019; Viertiö et al. 2009) and greater distance to public transportation infrastructures with longer duration of untreated psychosis (Malinowski et al. 2023). Care is not located solely in destinations or services, but emerges through the ordinary socio‐material relations that shape people's capacities to act, relate with others and engage with the city. This interpretation also has implications for debates on mobility justice (Sheller 2018).

In the light of our findings, mobility justice has to be understood in terms of how these infrastructures support people's capacity to benefit from mobility in everyday life. Beyond questions of access, rights and the equitable distribution of mobility opportunities, bus infrastructures also shape people's capacities to feel safe, regulate emotions and symptoms, rebuild confidence and gradually reconnect with urban life. For those living with psychosis or mental health difficulties, these infrastructures contribute to processes of becoming well. Justice, therefore, concerns whether they cultivate the capacities through which mobility becomes meaningful, and how their appropriation contributes to recovery and wellbeing. Bazile's experience illustrates this well: the disruption caused by the changing bus network shows clearly how motility is relational and fragile, not a stable personal attribute. His competence is not simply incorporated in him; it depends on the continuity and legibility of the infrastructure. When the network changes, his confidence and capacity to use it are affected. Bus infrastructures do not merely provide access; they actively sustain or, when disrupted, undermine the capacities through which people engage with the city and enact recovery.

Taken together, these findings suggest that buses should be understood not simply as transport services but as ordinary urban infrastructures through which recovery is continually negotiated. It thus becomes apparent that buses—and by extension public transportation infrastructures—can be seen as integral to the viability of community‐based mental health care, sustaining both logistical and affective dimensions of recovery. Yet, public transport infrastructures are rarely recognised as infrastructures of care—neither by local governments nor by care institutions. As Joelsson et al. note, ‘[r]eframing public transport as an infrastructure of care is also a way to address who is cared for and who is not cared for by public transport infrastructures’ (Joelsson et al. 2025, 10) and financial issues are particularly revealing in this regard: debates about fares, subsidies and eligibility criteria crystallise prevailing assumptions about what public transport is, what and who it is meant to serve and under what conditions support should be provided. As in most cities, public transportation is not free in Lausanne, Switzerland. Reduced fares and subsidies for public transport do, of course, exist, and they are largely structured around economic vulnerability, with eligibility tied to categories assumed to face financial pressure, such as students and seniors. Although substantial public transport discounts related to health issues are available, they still mainly operate through economically defined criteria^4^. In addition, obtaining DI status can be complex and lengthy, especially during periods of poor mental. Moreover, recovery‐oriented care may conflict with DI registration.

Recognising public transport as an infrastructure of care therefore shifts attention beyond questions of access and affordability towards the everyday conditions through which care is enacted. It foregrounds attention to addressing the experiential, affective and relational dimensions of mobility that shape mental wellbeing and recovery. Drawing on our empirical work, we argue that promoting buses and public transport as infrastructures of care requires more than fare‐free access—although removing fares can substantially reduce material and affective barriers. It calls for additional measures: mental health awareness training for drivers and ticket inspectors; the implementation of discreet communication tools such as the ‘Hidden Disability Sunflower’^5^ lanyard; and initiatives to counter stigma on buses and in transit spaces. Such interventions, when embedded within a wider ecology of care‐oriented urban initiatives—safe and welcoming third places, calm shopping hours, accessible green spaces, etc.—can help cultivate cities that are more habitable for those navigating early psychosis and other forms of mental distress and support processes of becoming well.

## Author Contributions

**Marc Winz:** funding acquisition, supervision, methodology, investigation, formal analysis, writing – original draft. **Ola Söderström:** conceptualisation, project administration, funding acquisition, writing – original draft. **Aurora Ruggeri:** methodology, investigation, formal analysis, writing – review and editing. **Jérôme Favrod:** conceptualisation, funding acquisition, writing – review and editing. **Jules Brischoux:** investigation. **Anastasie Jordan:** investigation. **Lilith Abrahamyan Empson:** conceptualisation, funding acquisition, investigation, writing – review and editing. **Philippe Conus:** conceptualisation, project administration, funding acquisition, writing – review and editing.

## Funding

This study was funded by the Swiss National Science Foundation (Grant 212633).

## Ethics Statement

Ethical approval was obtained from the local ethics committee (Commission cantonale (VD) d'éthique de la recherche sur l'être humain) protocol no. 2022‐02257. All participants were informed about the aims of the research and gave written consent prior to participation.

## Conflicts of Interest

The authors declare no conflicts of interest.

## Data Availability

The data supporting this study will be made available at the end of the project. In accordance with ethical and consent requirements, only the data from participants who have explicitly provided consent for further reuse will be shared.

## Endnotes

^1^
Much of the recent knowledge on mental health and transport stems from applied or policy‐oriented work – often published as reports commissioned by transport infrastructure services.

^2^
A comparable association has been found in the context of depression, where subjective limited access to the public transportation system was significantly associated with risk of depression in older adults, while objective measures were not (Matsumoto et al. 2025).

^3^
Gatrell (2013) expands the idea of therapeutic landscape to mobile dynamics in an influential paper where he suggests the notion of ‘mobile landscapes’. However, to the best of our knowledge, this notion has not been applied in the context of everyday uses of urban public transportation, but rather with regard to walking or travelling.

^4^
In Switzerland, reduced fares are granted to individuals who receive Disability Insurance (DI) and supplementary benefits (EL) only. The supplementary benefits “provide assistance in cases where the [DI] pensions and income do not cover the minimum living costs” (Information Center OASI/DI n.d.).

^5^
The Hidden Disability Sunflower is an increasingly adopted international initiative that provides a tool (a card and lanyard) for persons living with a disability or a condition to share it and/or to ask for assistance.
